# The direct-medical costs associated with interferon-based treatment for Hepatitis C in Vietnam

**DOI:** 10.12688/wellcomeopenres.15408.2

**Published:** 2020-09-11

**Authors:** Huyen Anh Nguyen, Graham S. Cooke, Jeremy N. Day, Barnaby Flower, Le Thanh Phuong, Trinh Manh Hung, Nguyen Thanh Dung, Dao Bach Khoa, Le Manh Hung, Evelyne Kestelyn, Guy E. Thwaites, Nguyen Van Vinh Chau, SEARCH Investigators, Hugo C. Turner

**Affiliations:** 1 Oxford University Clinical Research Unit, Ho Chi Minh, Vietnam; 2 Division of Infectious Diseases, Imperial College London, London, UK; 3 Centre for Tropical Medicine and Global Health, Nuffield Department of Medicine, University of Oxford, Oxford, UK; 4 Hospital for Tropical Diseases, Ho Chi Minh, Vietnam

**Keywords:** interferon-based therapy, direct medical costs, cost analysis, hepatitis C, Vietnam

## Abstract

**Background:** Injectable interferon-based therapies have been used to treat hepatitis C virus (HCV) infection since 1991. International guidelines have now moved away from interferon-based therapy towards direct-acting antiviral (DAA) tablet regimens, because of their superior efficacy, excellent side-effect profiles, and ease of administration. Initially DAA drugs were prohibitively expensive for most healthcare systems. Access is now improving through the procurement of low-cost, generic DAAs acquired through voluntary licenses. However, HCV treatment costs vary widely, and many countries are struggling with DAA treatment scale-up. This is not helped by the limited cost data and economic evaluations from low- and middle-income countries to support HCV policy decisions. We conducted a detailed analysis of the costs of treating chronic HCV infection with interferon-based therapy in Vietnam. Understanding these costs is important for performing necessary economic evaluations of novel treatment strategies.

**Methods:** We conducted an analysis of the direct medical costs of treating HCV infection with interferon alpha (IFN) and pegylated-interferon alpha (Peg-IFN), in combination with ribavirin, from the health sector perspective at the Hospital for Tropical Diseases, Ho Chi Minh City, Vietnam, in 2017.

**Results:** The total cost of the IFN treatment regimen was estimated to range between US$1,120 and US$1,962. The total cost of the Peg-IFN treatment regimen was between US$2,156 and US$5,887. Drug expenses were the biggest contributor to the total treatment cost (54-89%) and were much higher for the Peg-IFN regimen.

**Conclusions:** We found that treating HCV with IFN or Peg-IFN resulted in significant direct medical costs. Of concern, we found that all patients incurred substantial out-of-pocket costs, including those receiving the maximum level of support from the national health insurance programme. This cost data highlights the potential savings and importance of increased access to generic DAAs in low- and middle-income countries and will be useful within future economic evaluations.

## Introduction

The World Health Organization (WHO) estimates that there are 71 million people living with chronic hepatitis C infection globally ^1^. Hepatitis C virus (HCV) is typically transmitted through intravenous drug use, unsafe injection practice and the transfusion of unscreened blood/blood products ^1^. There are six major HCV strains, genotypes 1–6, and the prevalence of each genotype varies significantly between regions ^1^. Currently, most data relate to the treatment of genotypes 1–4; few data exist regarding the treatment outcomes and costs of genotype 6 infection, which accounts for over 50% of HCV infections in Vietnam ^2^.

Interferon-based therapies have been used to treat HCV since 1991 ^3^. The original interferon (IFN) intramuscular injections had to be administered daily and were associated with poor cure rates and unpleasant side effects ^4^. Pegylated IFN (Peg-IFN) was first licensed in 2001 ^5^ and has improved pharmacokinetics, requiring only weekly injections. Additionally, Peg-IFN is more effective than IFN and is associated with fewer adverse effects ^6^. When used for 24–48 weeks with the anti-viral tablet ribavirin, Peg-IFN is associated with cure rates of 54–63% (depending on the infecting viral genotype) ^7^. In recent years, new oral direct-acting antivirals (DAAs) have been developed. DAA therapy requires a shorter duration of treatment (typically 12 weeks) and has superior cure rates to interferon-based therapy (>95%) ^8^. The tablets have almost no side effects and forgo the need for weekly injections ^9^. When they first emerged, DAA drugs were prohibitively expensive for many healthcare systems, making them unavailable in most low- and lower-middle-income countries. Whilst prices still restrict access in many settings, the situation is improving, with steep price reductions for DAAs, driven largely by increased competition from generic manufacturers and the issuing of voluntary licenses ^10^. In 2016, 86% of people starting HCV therapy worldwide received DAA drugs rather than interferon-based therapy ^11^. Recently, the WHO recommended that interferon-based therapy should no longer be used where DAA drugs are available ^8^.

In 2016, the WHO released the first Global Health Sector Strategy on viral hepatitis with a goal of eliminating viral hepatitis as a public health threat by 2030 ^12^. The specific goals set for hepatitis C were that 80% of patients are treated, along with a 90% reduction in the incidence and a 65% reduction in HCV related mortality ^12^. In 2015, only 7% of the 71 million people living with chronic HCV infection were treated ^12^. Therefore, access to treatment needs to expand if the elimination goals are to be achieved.

Although improving, the global scale-up of DAA treatment has been markedly uneven, with a handful of countries (e.g. Egypt, China) accounting for the majority of the increase in uptake ^11^. A WHO analysis of country experiences of DAA scale-up shows that, while access to affordable treatment is important, countries also need a strong government response, including national plans for preventing, diagnosing and treating HCV, and adequate financing to roll out and sustain HCV services ^11, 13^. For this to occur, it is vital to have a detailed understanding of the cost and cost-effectiveness of the different treatment options available in low- and middle-income countries ^14, 15^.

We conducted a detailed analysis of the costs of treating chronic HCV with the pre-existing standard of care in Vietnam, IFN and Peg-IFN therapy. Since 2016, Vietnam has started to move away from interferon-based therapy towards DAA treatment regimens (in keeping with WHO guidelines). In June 2019, DAAs started to be covered by the national health insurance programme (NHI) ^16^. Consequently, due to its side effect profile and the increasing availability of DAAs, interferon-based therapy is becoming more infrequently used in Vietnam. However, data on the costs of interferon-based therapy are still essential for conducting accurate economic evaluations of DAA treatment, as interferon-based treatment will likely be the comparator ( Box 1) within the analysis.

**Box 1. Glossary.**

**Table:** 

**Catastrophic health expenditure**	When the medical expenditure of a household exceeds a certain level of capacity such that the household has to cut down on necessities (such as food, clothing, and their children's education).
**Comparator**	Within an economic evaluation, the new intervention being investigated is compared to a comparator. The comparator generally reflects the current clinical practice.
**Direct costs**	The costs related to the goods, services and resources consumed to implement and access healthcare.
**Direct medical costs**	The costs directly related to the use of medical services/resources (such as physician services, diagnostic tests and drugs).
**Direct non-medical costs**	The costs related to the consumption of non-medical resources (such as transportation to the health facility, food expenses and accommodation).
**Health sector perspective**	A perspective that only includes the costs associated with the health sector, such as the costs covered by the national health insurance programme and the patient’s copayment for the medical services.
**National health insurance programme (NHI)**	An insurance programme managed by the government that helps patients pay for medical services.
**Out-of-pocket payment**	The medical expenses incurred by patients that do not get reimbursed by insurance programmes. The out-of-pocket payment is usually equal to the price of the medical services/drugs in question multiplied by the patient’s co-payment rate.
**Patients co-payment rate**	The proportion of the total billed healthcare costs that insured patients pay. For example, if the insurers pay 80%, the remaining 20% will be paid by the patient.
**Perspective**	The viewpoint adopted for deciding which types of costs and health benefits are to be included within an economic evaluation.
**Productivity costs (indirect costs)**	Represent the value of the productivity losses that result from illness, treatment, or premature death.
**Societal perspective**	A perspective that includes all the costs associated with an intervention/healthcare, regardless of whom they are incurred by, i.e. this includes the health systems and patient’s direct medical costs, direct non-medical costs and indirect costs.
**Universal health coverage**	All individuals have access to good quality healthcare services without facing financial hardship.

## Methods

### Study location

Vietnam is a lower-middle-income country in Southeast Asia with a population of over 95 million people ^17^, and a 2017 GDP per capita of US$2365 ^18^. The seroprevalence of HCV in the general population has been estimated to be between 1 and 4.7% ^19, 20^, which is high relative to other countries in the region. In Vietnam, genotypes 1 and 6 predominate ^19, 21^. These genotypes are considered hardest to treat with interferon-based therapies and both genotypes 1 and 6 require prolonged treatment courses (48 weeks as opposed to 24 weeks) ^22^.

The Hospital for Tropical Diseases (HTD) in Ho Chi Minh City is the major referral hospital for infectious diseases in the south of Vietnam. Our cost estimation was performed in the context of the HTD in 2017.

### The resources and services required for HCV treatment

The Ministry of Health (MoH) approved four interferon-based treatments within their first HCV treatment guidelines in 2013: IFN α-2a, IFN α-2b, Peg-IFN α-2a and Peg-IFN α-2b ^23^. To enhance treatment efficacy, each of these injection-based treatments is combined with the antiviral tablet ribavirin ( Table 1). In late 2016, the MoH published an updated treatment guideline for Hepatitis C, in which the recommended treatments were Peg-IFN and DAAs. Although standard IFN was no longer included as a recommended treatment, it remained on the list of medicines covered by the national health insurance programme ( Box 1). From the end of 2016, IFN was no longer used at HTD. However, as it was still used in other hospitals in Vietnam, we have included cost analysis related to IFN treatment within this paper.

**Table 1.** The Vietnam MoH treatment guidelines for HCV drugs.

Name of drugs	Dose
IFN α-2a	3 million IU three times per week
IFN α-2b	3 million IU three times per week
Peg-IFN α-2a	180 μg once per week
Peg-IFN α-2b	1.5 μg/kg once per week
Ribavirin	Genotype 1/4/6: 1000mg per day
	Genotype 2/3: 800mg per day

Based on the HCV guidelines from the MoH in 2013 ^23^. IU, international unit.

We estimated the quantity of the drugs required for an average treatment based on the recommended dosages within the 2013 HCV treatment guidelines from the MoH ( Table 1) ^23^. The dosage of Peg-IFN α-2b was calculated assuming an average body weight of 58 kilograms for men and 50 kilograms for women ^24^.

The utilisation of the other resources and services (such as the medical tests) required to provide HCV treatment were based on the MoH 2013 treatment guidelines ^23^. These recommend that a patient should visit the outpatient clinic once prior to treatment, every four weeks during treatment and once after ending treatment. A summary of the required medical tests at these different stages of treatment is shown in Table 2. Following the hospital’s classification, the tests were grouped into seven different classes. The duration of treatment depends on the genotype of HCV: 24 weeks for genotypes 2/3 and 48 weeks for genotypes 1/4/6 ^23^. Both of these regimens were considered in our analysis.

**Table 2.** A summary of the recommended medical tests within the Vietnam Ministry of Health (MoH) hepatitis C virus (HCV) treatment guidelines.

Name of required tests		Before treatment	During treatment	After treatment
**Group 1:** **Electrocardiogram**	Electrocardiogram	Yes	No	No
**Group 2: Ultrasound**	Abdominal ultrasound		Every 12 weeks	
	Fibro-scan		No	
**Group 3: X-ray**	Chest X-ray		No	
**Group 4: Blood tests**	Full blood count		Every 4 weeks	
	The international normalized ratio		No	
	Prothrombin		Every 12 weeks	
**Group 5:Immunoassay**	Alpha-fetoprotein			
	Free thyroxine			
	Thyroid-Stimulating Hormone			
	Hepatitis B surface antigen		No	
	Human immunodeficiency virus			
**Group 6: Biochemical tests**	Electrolytes			
	Albumin			
	Bilirubin			
	Creatinine (urine and blood)		Every 4 weeks	
	Urea		No	
	Alanine transaminase		Every 4 weeks	
	Aspartate aminotransferase		No	
	Gamma-glutamyl transferase		No	
**Group 7: Molecular biology** **tests**	HCV-RNA viral load test		Every 8 weeks	Yes
	HCV genotype real-time PCR		No	No

Before treatment, certain tests are required to assess disease severity and to ensure that treatment can be safely tolerated. During treatment, monitoring tests are required every 4, 8 or 12 weeks to assess treatment response and drug side effects. After treatment, the HCV-RNA viral load test is repeated to assess treatment response. This is based on the 2013 HCV treatment guidelines from the MoH ^23^.

### Cost estimation and outputs

Our cost analysis estimated the direct medical cost of HCV treatment from the health sector perspective ( Box 1). The direct non-medical costs and indirect costs ( Box 1) were not quantified.

The identified resources and services ( Table 1 and Table 2) were costed based on the services and drug unit price list of HTD in 2017 relating to those covered by the national health insurance programme ^25^. One exception to this was the costs relating to the IFN drugs which were obtained from a 2017 report from the Drug Administration of Vietnam ^26^.

The main output was the total cost of the different treatments, stratified by the three main cost components: the cost of the drugs, the cost of the medical tests and the costs related to the clinical consultation fees.

All costs were converted to US dollars (US$) following the average 2017 exchange rate where 22,370 Vietnamese dong (VND) equal 1 US$ ^27^.

## Results

The total cost of the IFN treatment regimen was estimated to range between US$1,120 and US$1,962 and the total cost of the Peg-IFN treatment regimen between US$2,156 and US$5,887 ( Table 3). The cost of treating genotypes 1/4/6 (which require a 48-week treatment regimen) was substantially higher than the cost of treating genotypes 2/3 (which require a 24-week regimen). The cost was not exactly double, due to the different dosages of ribavirin used for the different genotypes ( Table 1 and Table 3) and the fact that the pre-and post-treatment tests are identical.

**Table 3.** The estimated cost of the different regimens.

Treatment	Drugs (US$)		Tests (US$)	Consultation fees (US$)	Total treatment cost (US$)
	Total	Average per week			
	Genotype 2/3: 24-week treatment regimen				
**IFN α-2a + ribavirin**	619.17 – 655.17	25.79 - 27.30	498.18	13.95	1,155.29 – 1,167.31
**IFN α-2b + ribavirin**	607.75 – 619.77	25.32 - 25.82	498.18	13.95	1,119.88 – 1,131.90
**Peg-IFN α-2a +** **ribavirin**	1,644.14 – 2,617.89	68.50 - 109.07	498.18	13.95	2,156.27 – 3,130.02
**Peg-IFN α-2b +** **ribavirin**	1,680.07 – 1,967.78	70.00 - 81.99	498.18	13.95	2,192.20 – 2,470.91
	Genotype 1/4/6: 48-week treatment regimen				
**IFN α-2a + ribavirin**	1,325.52 – 1,333.78	27.61 - 27.79	613.24	24.41	1,953.84 – 1,961.90
**IFN α-2b + ribavirin**	1,254.71 – 1,262.97	26.14 - 26.31	613.24	24.41	1,882.83 – 1,892.09
**Peg-IFN α-2a +** **ribavirin**	3,327.48 – 5,259.20	69.32 - 109.57	613.24	24.41	3,955.60 – 5,887.32
**Peg-IFN α-2b +** **ribavirin**	3,375.89 – 3,950.73	70.33 - 82.30	613.24	24.41	4,003.43 – 4,578.85

The range in the costs for a given regimen is due to the variation in the costs of the different brands of the drugs and the different dosages (minimum and maximum values are shown in Table 4). Costs are in 2017 prices.

We estimated the costs of the three main components of HCV treatment: the drugs, medical tests and clinical consultation fees. The costs of the drugs contributed between 54–89% to the total treatment cost and were much higher for the Peg-IFN regimen. These were shown as a range because the exact price varies depending on which brand is used ( Table 4). This variation was most significant for Peg-IFN α-2a. The cost of the ribavirin only represented 2–8% of the costs relating to the drugs.

**Table 4.** The assumed input and unit cost for the direct medical cost of interferon-based treatment for Hepatitis C in Vietnam.

Item		Total Quantity		Unit cost (VND)	Unit cost (US$)
		Genotype 2/3	Genotype 1/4/6		
**Drug**					
**IFN α-2a**	Feronsure (3×10 ^6^ IU)	216×10 ^6^	432×10 ^6^	189000	8.32
**IFN α-2b**	Superferon (3×10 ^6^ IU)	216×10 ^6^	432×10 ^6^	178000	7.84
**Peg-IFN α-2a**	Pegasys (135 μg)	4320	8640	1797313	79.14
	Pegasys (135 μg) new version	4320	8640	2327195	102.48
	Pegasys (180 μg) old version	4320	8640	1400000	61.65
	Pegasys (180 μg) new version	4320	8640	1950000	85.87
	Pegnano (180 μg) old version	4320	8640	1750000	77.06
	Pegnano (180 μg) new version	4320	8640	1500000	66.05
**Peg-IFN α-2b**	Peg-intron (50 μg) ^a^	Man: 2088 Woman:1800	Man: 4176 Woman: 3600	1014860	44.69
	Peg-intron (80 μg) ^a^	Man: 2088 Woman:1800	Man: 4176 Woman: 3600	1639400	72.19
	Peg-intron redipen (100 μg) ^a^	Man: 2088 Woman:1800	Man: 4176 Woman: 3600	2058000	90.62
**Ribavirin**	Barivir (400 mg)	403200	336000	2900	0.13
	Barivir (500 mg)	403200	336000	3900	0.17
**Medical tests**					
***Group 1: Electrocardiogram***					
** Electrocardiogram**		1	1	45900	2.02
***Group 2: Ultrasound***					
** Abdominal ultrasound**		3	5	49000	2.16
** Fibro-scan**		1	1	79500	3.50
***Group 3: X-ray***					
** Chest X-ray**		1	1	69000	3.04
***Group 4: Blood tests***					
** Full blood count**		1	1	44800	1.97
** The international normalized ratio**		1	1	12300	0.54
** Prothrombin**		3	5	61600	2.71
***Group 5: Immunoassay***					
** Alpha-fetoprotein**		3	5	90100	3.97
** Free thyroxine**		3	5	63600	2.80
** Thyroid-stimulating Hormone**		3	5	58300	2.57
** Hepatitis B surface antigen**		1	1	712000	31.35
** Human immunodeficiency virus**		1	1	319700	14.08
***Group 6: Biochemical tests***					
** Electrolytes**		1	1	28600	1.26
** Albumin**		1	1	21200	0.93
** Bilirubin**		1	1	21200	0.93
** Creatinine (urine)**		6	12	15900	
** Creatinine (blood)**		7	13	21200	0.93
** Urea**		1	1	21200	0.93
** Alanine transaminase**		1	1	21200	0.93
** Aspartate aminotransferase**		7	13	21200	0.93
** Gamma-glutamyl transferase**		1	1	19000	0.84
***Group 7: Molecular biology tests***					
** HCV-RNA viral load test**		5	8	1310000	57.69
** HCV genotype: Real time PCR**		1	1	1550000	68.25
**Consultation**					
** Clinical consultation fees**		8	14	39000	1.72

^a^The dosage is prescribed following average body weight for men is 58 kg and for women is 50 kg ^29^. Costs are in 2017 prices. IU, international unit

**Figure 1.**
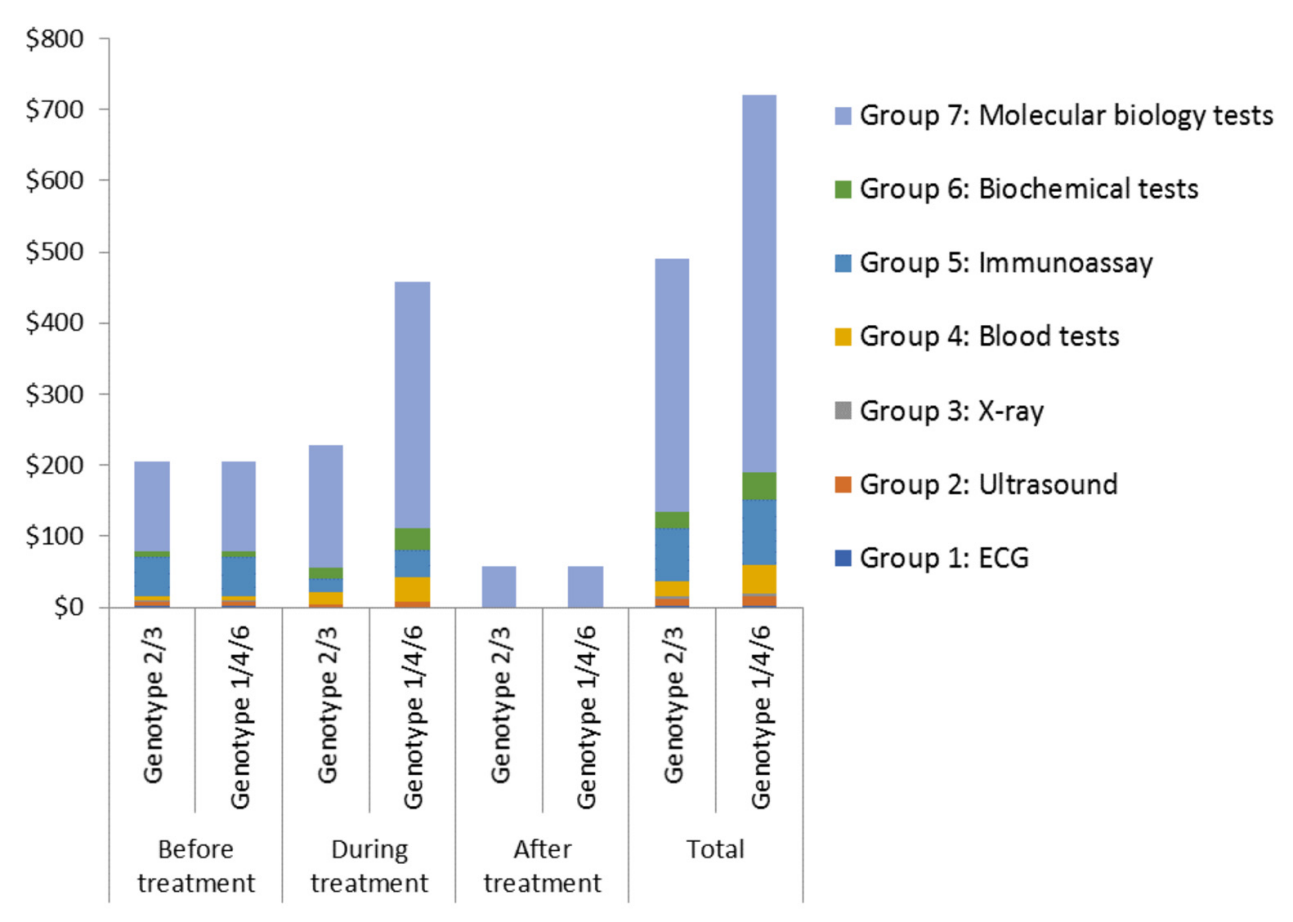
The cost of the medical tests associated with treating genotypes 2/3 (24-week treatment regimen) and genotypes 1/4/6 (48-week treatment regimen). A summary of the recommended medical tests at different stages of hepatitis C virus treatment is shown in Table 2. The unit costs of the different types of tests are shown in Table 3. Costs are in 2017 prices.

The costs of the medical tests were also notable (US$498 for treating genotypes 2/3 and US$613 for treating genotypes 1/4/6) ( Figure 1). The HCV-RNA viral load tests accounted for the majority of this (approximately 70%). The costs relating to the tests used pre- and post-treatment were the same for both genotype groups ( Figure 1). However, the costs of the tests used during the treatment were double for the 48-week treatment regimen compared to the 24-week regimen (genotypes 1/4/6 vs genotypes 2/3) ( Table 2 and Figure 1).

## Discussion

Treating HCV in Vietnam with IFN or Peg-IFN results in significant direct medical costs. These costs are particularly high because the genotypes that are most prevalent (1 and 6) require a prolonged (48 weeks) duration of therapy ^28^. The drug-related costs contributed the most (54–89%) to the total treatment cost ( Table 3). The drug-related costs for treating HCV genotypes 1/6 were approximately US$25 per week for IFN plus ribavirin and between US$68-109 per week for Peg-IFN plus ribavirin. The costs of the medical tests and monitoring also contributed notably to the total treatment cost (10–44%) and were related to the duration of the treatment.

The results relate to the cost of a treatment regimen and not the cost per patient cured. Although the cost of treatment with IFN is cheaper, it is less likely to cure the patient successfully and the costs associated with treatment failure can be significant. Consequently, IFN treatment is rarely used in Vietnam.

Studies in neighboring countries have reported the cost of Peg-IFN using the same doses. One study from Thailand reported that the Peg IFN-2a/2b and ribavirin for treating HCV genotypes 1/6 cost US$90 per week (2013 prices) ^30^, which is similar to our finding. Another study reported that in China ^31^, the Peg IFN-2a and ribavirin for treating HCV genotype 1, cost US$174 per week (2016 prices), almost double our estimate.

These cost estimates relate to a standard treatment (based on MoH guidelines). It should be noted that in practice there will be variations in these treatment costs and resources utilized, such as for those not finishing the treatment regimen, experiencing treatment failure or those with co-infections such as HIV.

### Payment from the national health insurance programme

In Vietnam, the proportion of the population covered by the NHI as of December 2016 is estimated to be 81.7% ^32^. However, even the patients covered by the NHI can incur significant out-of-pocket payments ( Box 1) for HCV treatment. For the drug costs relating to HCV treatment, the patient’s out-of-pocket payment will be based on the price of the drug and the patient’s co-payment rate (the proportion of the billed costs that insured patients pay) ( Equation 1).

      Equation 1: Out-of-pocket payment for the drugs = Price of the drug × Patient’s co-payment rate 

**Figure 2.**
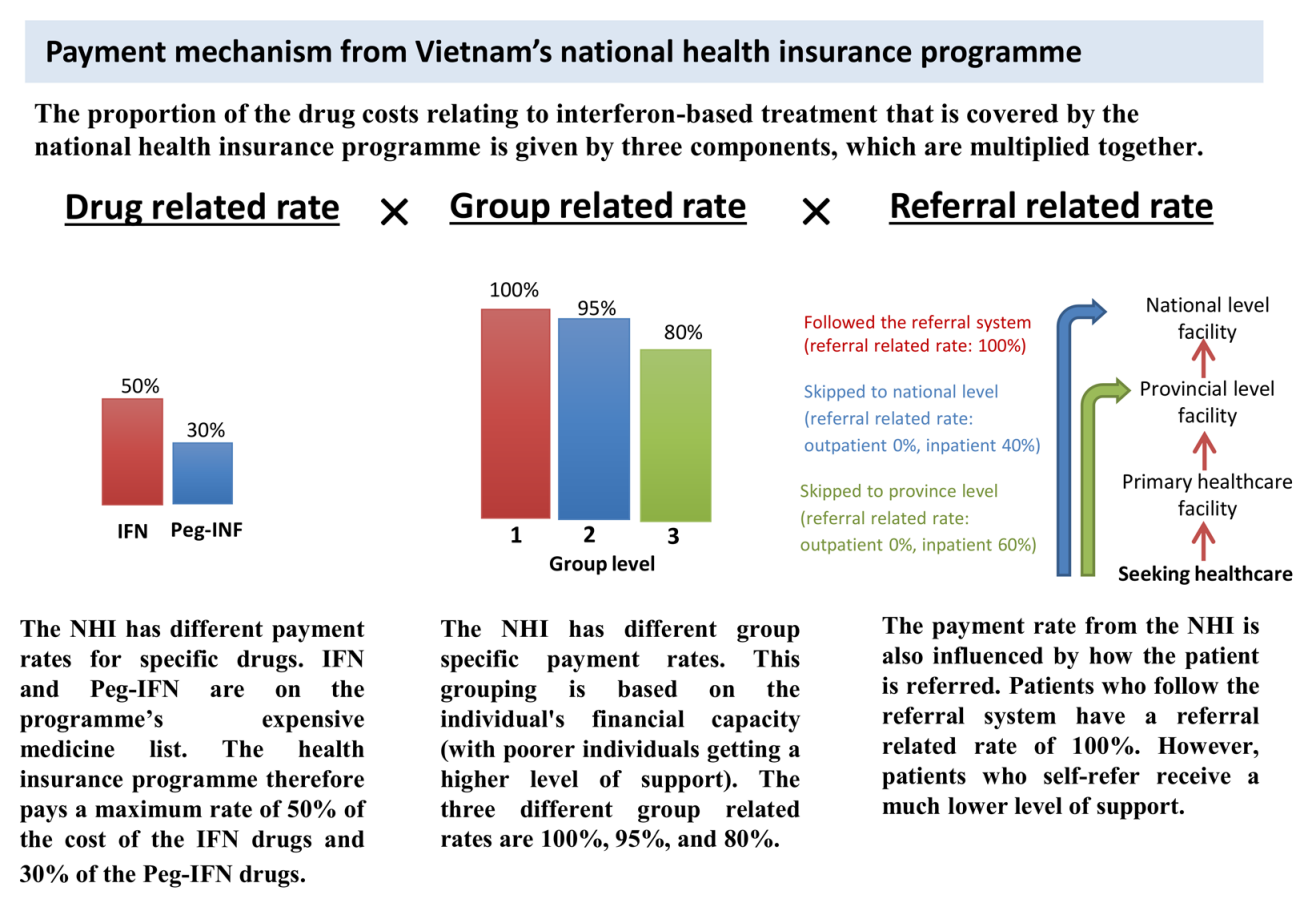
Summary of the payment mechanism for the drug costs relating to interferon-based treatment from the national health insurance programme. Information adapted from 16, 33. NHI: National health insurance programme.

The patient’s co-payment rate is what is remaining after subtracting the proportion covered by the NHI ( Equation 2). In the case of these drug costs, the proportion covered by the NHI ( Equation 3) is given by three components (the drug related rate, group related rate and referral related rate). These are outlined in Figure 2.

      Equation 2: Patients co-payment rate = 1 – proportioncovered by the NHI

      Equation 3: Proportion covered by the NHI = Drug relatedrate × Group related rate × Referral related rate

**Figure 3.**
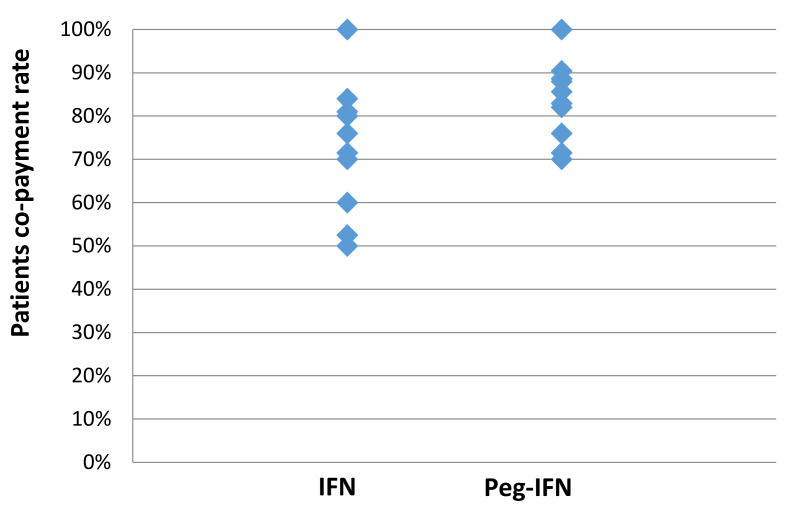
The possible co-payments required by patients for the drug costs relating to interferon-based treatment. The values summarize the different potential patient co-payment rates for the drug costs relating to interferon-based treatment (based on Figure 2 and Equation 2). Note, these only pertain to the drugs and not the other resources/services.

Based on these rates, even with the maximum level of insurance cover, patients still have to pay 50% of the cost of the IFN drugs and 70% of the cost of the Peg-IFN drugs (See Drug related rate within Figure 2) ^16^. Furthermore, if patients attend the HTD without a formal referral from their primary health care facility, they have to pay for the full cost of the treatment (as though uninsured) (See Referral related rate within Figure 2 and Figure 3) ^33^. The payment mechanism within the NHI is the same for the non-drug costs, but the drug related rates are superseded by the relevant non-drug rates (see Equation 3).

### Catastrophic health expenditures

In Vietnam, the average income in 2016 was US$136 per month ^34^. In comparison, the estimated costs of HCV treatment with IFN or Pre-IFN ranged between US$200 and US$480 per month ( Table 3). Given that even patients receiving the maximum level of support from the NHI incur substantial out-of-pocket payments for these treatments ( Figure 3), it is likely that many patients will have been unable to afford interferon-based HCV treatment. This financial barrier may have led to many patients not being able to access treatment, as well as what is known as “catastrophic health expenditures” ( Box 1) (this is when medical spending of a household reaches a point such that the household has to cut down on necessities (such as food, clothing, and their children's education)) ^35^. A variety of different thresholds are used to define this, such as 25% of total household expenditure/income or 40% of a household’s non-subsistence expenditure ^35, 36^. Regardless of the exact threshold used, our results indicate that interferon-based treatments were causing catastrophic health expenditures in Vietnam. The importance of reducing such financial barriers is recognised in the Sustainable Development Goals ^37^.

### The move towards using DAAs

DAAs were initially very expensive, thereby restricting their use to high-income countries ^38^. However, the emergence of low-cost generic versions of the drugs, has led to steep price reductions. It has been estimated that widespread access to combinations of HCV DAAs is feasible, with potential target prices approximately US$50–$250 per person for a standard 12-week treatment course, significantly cheaper than the longer treatment regimens with IFN and Peg-IFN A recent Sstudiesy ^39– 41^ suggested that widespread access to combinations of HCV DAAs is feasible, with potential target prices of US$100–$250 per person for a standard 12-week treatment course, significantly cheaper than the longer treatment regimens with IFN and Peg-IFN ^39, 1, 2^. The new DAA drugs are ‘pangenotypic’, meaning they are similarly efficacious for different genotypes, removing the need for expensive genotype testing in specialist labs or prolongation of therapy for the predominant strains in Vietnam. Because DAA treatment regimens are shorter, with fewer side effects, there will also be cost savings associated with the medical monitoring and consultations required during treatment compared to interferon-based therapy ( Figure 1).

A further potential advantage of DAAs is that they may allow the possibility of decentralising hepatitis C treatment to primary health care facilities in some settings ^42^, which could also bring additional cost savings ^43^. This benefit will depend on the local health system and needs further investigation.

This cost data related to interferon-based therapy indicates that switching to generic DAAs may lead to potential cost savings.

The most recent Vietnamese MoH treatment guidelines (released in 2016) recommend DAAs as the first-line therapy ^44^. However, the costs of DAA drugs only became subsidised by the NHI in June 2019.

### Limitations

Our study has several limitations, for example, we focused on quantifying only the direct medical costs of HCV treatment. This includes the costs covered by the insurance system and the patient’s co-payment ( Box 1). However, the direct non-medical costs (such as the patient's travel costs) and the patient’s productivity costs (indirect costs) were not quantified. The total cost of HCV treatment under the societal perspective ( Box 1) would therefore be even higher. It was also not possible to capture the costs associated with the specific side-effects of IFN/Peg-IFN treatment. In our study, we focused only on patients with HCV infection; in practice, the prevalence of co-infections with other hepatotropic viruses or HIV can be high, and this is likely to influence the treatment costs.

Our analysis was performed in the context of the HTD in Ho Chi Minh City, which is a large hospital specialising in infectious disease. Whilst it is possible that there may be some minor variations in costs in other provinces in Vietnam, as the costs of both healthcare services and drugs are regulated centrally by the NHI department and MoH, our cost estimates are likely to be robust. Although the precise results and cost estimates of our study are not directly generalizable to other countries, they are consistent with reports from neighbouring countries, such as Thailand ^30^. It is important that further HCV treatment costing studies are conducted in other low- and middle-income countries, particularly relating to the use of DAAs.

The cost estimates were predominantly based on the price lists from HTD relating to 2017. However, it is possible that the costs, particularly those relating to the drugs, will vary over time.

We have focused on conducting a costing study, hence further evaluation regarding the cost-effectiveness of the different treatments is required.

## Conclusion

A deeper understanding of the costs of the different treatment options is vital for supporting HCV policy decisions. Currently, there are few costing studies and economic evaluations of HCV treatment in low- and middle-income countries ^15, 45, 46^.

We found that treating HCV with IFN or Peg-IFN results in significant direct medical costs. We estimated that a 48-week Peg-IFN treatment regimen costs between US$3,956–5,887 in Vietnam. The majority of this figure relates to the cost of the drugs.

Although the role of interferon-based therapy is diminishing, this cost data provides a foundation for evaluating the economic benefits and cost-effectiveness of switching to using DAAs.

Of concern, we found that even patients receiving the maximum level of support from the NHI incur substantial out-of-pocket costs for their HCV treatment ( Figure 3). Consequently, many patients will not be able to afford the IFN or Peg-IFN treatments, leading to “catastrophic health expenditures” ( Box 1). This raises important issues regarding the health insurance payment mechanism for HCV patients. Once newer interferon-free regimens are included in the government’s insurance coverage, out-of-pocket expenses for patients could be reduced, but details of how this will be managed are not yet available. Crucially, minimising costs to patients will be an important part of reaching the ambitious 2030 treatment targets ^12^.

## Data availability

All data underlying the results are available as part of the article and no additional source data are required.
