# Supplementary material for: The direct-medical costs associated with interferon-based treatment for Hepatitis C in Vietnam
Source: Wellcome Open Res. 2020 Sep 11;4:129. Originally published 2019 Sep 4. [Version 2] doi: 10.12688/wellcomeopenres.15408.2 (PMC7372532; doi:10.12688/wellcomeopenres.15408.2)
Supplement: Peer review history

# Peer review: Reviewer response for version 1

**DOI:** 10.21956/wellcomeopenres.16845.r39396

**Published:** 2020-07-20

**Authors:** Rodolfo Castro

**Affiliations:** 1 Oswaldo Cruz Foundation, FIOCRUZ, Rio de Janeiro, Brazil; 2 Federal University of the State of Rio de Janeiro, UNIRIO, Rio de Janeiro, Brazil

In summary, the research can be considered a critical previous step towards improving access to DAAs in Vietnam. It was concluded that treating HCV with IFN or Peg-IFN results in high direct medical costs. DAAs are subsidised by the local National health insurance programme from June 2019.

 Some discretionary suggestions to improve the paper´s quality are listed below:

 It would be useful to have a supplemental spreadsheet that could be used as a parameter for further economic evaluations; this spreadsheet could have citations (or weblinks to the sources used) to allow quick update and checking by future researchers.

 An update regarding the actual use of IFN-based regimens for Hepatitis C treatment in Vietnam would be useful.

 Authors’ could also discuss, as another potential advantage of DAAs, the possibility of decentralising Hepatitis C treatment to primary health care ^1^; this could bring additional savings if associated with the use of effective generic drugs ^2^.

Is the work clearly and accurately presented and does it cite the current literature?

Yes

If applicable, is the statistical analysis and its interpretation appropriate?

Not applicable

Are all the source data underlying the results available to ensure full reproducibility?

Partly

Is the study design appropriate and is the work technically sound?

Yes

Are the conclusions drawn adequately supported by the results?

Yes

Are sufficient details of methods and analysis provided to allow replication by others?

Yes

Reviewer Expertise:

Public Health; Health Technology Assessment; Epidemiology

I confirm that I have read this submission and believe that I have an appropriate level of expertise to confirm that it is of an acceptable scientific standard.

## References

[1] : Effectiveness of implementing a decentralized delivery of hepatitis C virus treatment with direct-acting antivirals: A systematic review with meta-analysis. *PLoS One*.2020;15(2) : 10.1371/journal.pone.0229143 e0229143 10.1371/journal.pone.0229143 32084187PMC7034833

[2] : Effectiveness of generic direct-acting agents for the treatment of hepatitis C: systematic review and meta-analysis. *Bull World Health Organ*.2020;98(3) : 10.2471/BLT.19.231522 188-197K 10.2471/BLT.19.231522 32132753PMC7047023

# Response

**Published:** 2020-08-31

**Authors:** Huyen Nguyen

**Affiliations:** Oxford University Clinical Reseach, Vietnam

In summary, the research can be considered a critical previous step towards improving access to DAAs in Vietnam. It was concluded that treating HCV with IFN or Peg-IFN results in high direct medical costs. DAAs are subsidised by the local National health insurance programme from June 2019.

Some discretionary suggestions to improve the paper´s quality are listed below:

It would be useful to have a supplemental spreadsheet that could be used as a parameter for further economic evaluations; this spreadsheet could have citations (or weblinks to the sources used) to allow quick update and checking by future researchers.

**Response:** We have included the full detail of the costs used within the analysis within Table 4.

An update regarding the actual use of IFN-based regimens for Hepatitis C treatment in Vietnam would be useful.

**Response: **We have added the following into the introduction.

*“We conducted a detailed analysis of the costs of treating chronic HCV with the pre-existing standard of care in Vietnam, IFN and Peg-IFN therapy. Since 2016, Vietnam has started to move away from interferon-based therapy towards DAA treatment regimens (in keeping with WHO guidelines). In June*

*2019, DAAs started to be covered by national health insurance ^32^. Consequently, due to its side effect profile and the increasing availability of DAAs, interferon-based therapy is becoming more infrequently used in Vietnam.  However, data on the costs of interferon-based therapy are still essential for conducting accurate economic evaluations of DAA treatment, as interferon-based treatment will likely be the comparator (* *Box 1* *) within the analysis.”*

Unfortunately, there is currently no data on the exact numbers receiving interferon-based therapy nationally.

Authors’ could also discuss, as another potential advantage of DAAs, the possibility of decentralising Hepatitis C treatment to primary health care 1; this could bring additional savings if associated with the use of effective generic drugs 2.

**Response: **We agree and really appreciate your suggestion. We have added the following with your suggested references:

*“A further potential advantage of DAAs is that they may allow the possibility of decentralising hepatitis C treatment to primary health care facilities in some settings [3], which could also bring additional cost savings [4]. This benefit will depend on the local health system and needs further investigation.”*

# Peer review: Reviewer response for version 1

**DOI:** 10.21956/wellcomeopenres.16845.r37603

**Published:** 2020-01-30

**Authors:** Josephine Walker

**Affiliations:** 1 Population Health Sciences, Bristol Medical School, University of Bristol, Bristol, UK

This article presents important information on the cost of treatment for Hepatitis C virus with the older drug regimens that have been in use since 1991 (IFN and Peg-IFN). Although new recommendations are to use direct acting antiviral (DAA) treatments when available, it is important to understand the baseline or previous cost of treatment to accurately estimate the impact of transitioning to new treatment regimens.

 There are a few points it would be helpful to address in the discussion: 

- The results include a range of uncertainty in the cost of the drugs, based on different brands available, but there is no uncertainty or variation included in the cost of the tests or in the number of tests received per patient. Perhaps comment on whether all patients are expected to receive the same tests or if some will fail to complete treatment.
- The total cost per week and cost per month are referred to in the discussion, it would be clearer if these estimates are included in the results so that the reader doesn't have to calculate it themselves.
- The point about catastrophic health expenditure is interesting, but made me wonder whether it means patients aren't able to access treatment at all rather than spending beyond their means. Would this change with DAA treatment? Is there any information available about access to healthcare and how this varies according to the distribution of income in the country?
- Furthermore, the change to DAA drugs may result in changes to the number of tests and consultations required due to fewer side effects, which may be worth mentioning.
- It is stated that there are few costing studies and economic evaluations of HCV treatment in LMIC, but no references are included in the discussion to show the few that are available. Some are referenced earlier in the paper regarding IFN treatment, and there are published papers of economic evaluation of DAAs in Egypt and India (plus others in press).

Figure 2 - I think some text is needed to explain this figure as I couldn't follow it. Do the uses of the words "co-payment" vs "out-of-pocket payment" here match the definitions in Box 1? Perhaps converting this into a box instead of a figure would allow for additional explanation of how the co-payment mechanism works? In addition, it wasn't clear to me how the co-payment applies to non-drug costs, and this will also apply to the discussion of catastrophic health expenditures. 

 Minor changes including editorial points:

- Introduction first paragraph contains an extra close brackets at the end of line 7.
- Methods first paragraph refers to gross per capita income but cites a source of GDP per capita. Clarify if referring to GDP, GNI, or per capita income which is referred to in discussion with a different source cited.
- Table 3 footnote refers to itself, should this refer to Table 4?
- Table 4, please indicate which class of drugs each brand name refers to so that it can be cross-referenced with Table 3.
- Figure 3 caption refers to itself, should this refer to Figure 2?
- The reference for the cost of DAAs is fairly old, unfortunately there is not much published on this but MSF's press release on what price they pay may be helpful.
- Discussion states that cost data related to IFN supports economic case to increase access to DAAs. Perhaps rephrase to indicate that the cost data provides a foundation for evaluating the economic case to treat with DAAs, as the case for use of DAAs is not made in this paper. Or remove as this is stated later in discussion as well (bottom of page 9 column 1).

Is the work clearly and accurately presented and does it cite the current literature?

Yes

If applicable, is the statistical analysis and its interpretation appropriate?

Not applicable

Are all the source data underlying the results available to ensure full reproducibility?

Yes

Is the study design appropriate and is the work technically sound?

Yes

Are the conclusions drawn adequately supported by the results?

Partly

Are sufficient details of methods and analysis provided to allow replication by others?

Yes

Reviewer Expertise:

Evaluation of health interventions in low and middle income countries, particularly related to the impact and cost-effectiveness of scaling up treatment for Hepatitis C virus.

I confirm that I have read this submission and believe that I have an appropriate level of expertise to confirm that it is of an acceptable scientific standard.

# Response

**Published:** 2020-08-31

**Authors:** Huyen Nguyen

**Affiliations:** Oxford University Clinical Reseach, Vietnam

This article presents important information on the cost of treatment for Hepatitis C virus with the older drug regimens that have been in use since 1991 (IFN and Peg-IFN). Although new recommendations are to use direct acting antiviral (DAA) treatments when available, it is important to understand the baseline or previous cost of treatment to accurately estimate the impact of transitioning to new treatment regimens.

 There are a few points it would be helpful to address in the discussion: 

 The results include a range of uncertainty in the cost of the drugs, based on different brands available, but there is no uncertainty or variation included in the cost of the tests or in the number of tests received per patient. Perhaps comment on whether all patients are expected to receive the same tests or if some will fail to complete treatment.

**Response: **The costs were calculated in relation to a standard treatment (based on the treatment guidelines). There will be some variation and we have added the following to the discussion. 

*“These cost estimates relate to a standard treatment (based on MoH guidelines). It should be noted that in practice there will be variations in these treatment costs and resources utilized, such as for those not finishing the treatment regimen. experiencing treatment failure or those with co-infections such as HIV.”*

 The total cost per week and cost per month are referred to in the discussion, it would be clearer if these estimates are included in the results so that the reader doesn't have to calculate it themselves.

**Response:**  We agree that this would be clearer and have added a column for the average cost per week for the drug cost in Table 3.

 The point about catastrophic health expenditure is interesting, but made me wonder whether it means patients aren't able to access treatment at all rather than spending beyond their means. Would this change with DAA treatment? Is there any information available about access to healthcare and how this varies according to the distribution of income in the country?

**Response**: This is a very good point. We have added more in the text that the high costs may have meant that many patients were not accessing treatment, as well as leading to catastrophic health expenditure. Unfortunately, there is currently no information available about access to healthcare and how this varies according to the distribution of income in the country and we are planning to explore how it may change with DAA treatment in future work.

*“In Vietnam, the average income in 2016 was US$136 per month* *^34^* *. In comparison, the estimated costs of HCV treatment with IFN or Pre-IFN ranged between US$200 and US$480 per month (* *Table 3* *). Given that even patients receiving the maximum level of support from the national health insurance programme incur substantial out-of-pocket payments for these treatments (* *Figure 3* *), it is likely that many patients will have been unable to afford interferon-based HCV treatment. This financial barrier may have led to many patients not being able to access treatment, as well as what is known as “catastrophic health expenditures” ( Box 1) (this is when medical spending of a household reaches a point such that the household has to cut down on necessities (such as food, clothing, and their children's education))* *^35^* *. A variety of different thresholds are used to define this, such as 25% of total household expenditure/income or 40% of a household’s non-subsistence expenditure* *^35^* *^,^* *^36^* *. Regardless of the exact threshold used, our results indicate that interferon-based treatments were causing catastrophic health expenditures in Vietnam. The importance of reducing such financial barriers is recognised in the Sustainable Development Goals”*

 Furthermore, the change to DAA drugs may result in changes to the number of tests and consultations required due to fewer side effects, which may be worth mentioning.

**Response**: We have the added the following in the “ The move towards using DAAs” section

*“The new DAA drugs are ‘pangenotypic’, meaning they are similarly efficacious for different genotypes, removing the need for expensive genotype testing in specialist labs or prolongation of therapy for the predominant strains in Vietnam. Because DAA treatment regimens are shorter, with fewer side effects, there will also be cost savings associated with the reduced medical monitoring and consultations required during treatment compared to interferon-based therapy.”*

**

 It is stated that there are few costing studies and economic evaluations of HCV treatment in LMIC, but no references are included in the discussion to show the few that are available. Some are referenced earlier in the paper regarding IFN treatment, and there are published papers of economic evaluation of DAAs in Egypt and India (plus others in press).

**Response:** We have added appropriate references for this statement – including two systematic reviews.

 “Currently, there are few costing studies and economic evaluations of HCV treatment in low- and middle-income countries [REFS 1-3]”

**REF 1**: Chhatwal J, He T, Lopez-Olivo MA. Systematic review of modelling approaches for the cost effectiveness of hepatitis C treatment with direct-acting antivirals. Pharmacoeconomics. 2016;34(6):551-67.

**REF 2**: He T, Lopez‐Olivo M, Hur C, Chhatwal J. Systematic review: cost‐effectiveness of direct‐acting antivirals for treatment of hepatitis C genotypes 2‐6. Alimentary pharmacology & therapeutics. 2017;46(8):711-21.

**REF 3**: Chaillon A, Mehta SR, Hoenigl M, Solomon SS, Vickerman P, Hickman M, et al. Cost-effectiveness and budgetary impact of HCV treatment with direct-acting antivirals in India including the risk of reinfection. PloS one. 2019;14(6):e0217964.

 Figure 2 - I think some text is needed to explain this figure as I couldn't follow it. Do the uses of the words "co-payment" vs "out-of-pocket payment" here match the definitions in Box 1? Perhaps converting this into a box instead of a figure would allow for additional explanation of how the co-payment mechanism works? In addition, it wasn't clear to me how the co-payment applies to non-drug costs, and this will also apply to the discussion of catastrophic health expenditures. 

**Response:** We have changed Figure 2 to make it clearer and add more explanation of how the co-payment mechanism works within the text.

*“In Vietnam, the proportion of the population covered by the national health insurance programme (NHI) as of December 2016 is estimated to be 81.7% ^31^. However, even the patients covered by the NHI can incur significant out-of-pocket payments (Box 1) for HCV treatment. For the drug costs relating to HCV treatment, the patient’s out-of-pocket payment will be based on the price of the drug and the patient's co-payment rate (the proportion of the billed costs that insured patients pay) (Equation 1).*

*Equation 1: Out-of-pocket payment for the drugs = Price of the drug × Patient’s co-payment rate*

*The patient’s co-payment rate is what is remaining after subtracting the proportion covered by the NHI (Equation 2). In the case of these drug costs, the proportion covered by the NHI (Equation 3) is given by three components (the drug related rate, group related rate and referral related rate). These are outlined in Figure 2.*

*Equation 2: Patients co-payment rate = 1 – proportion covered by the NHI*

*Equation 3: Proportion covered by the NHI = Drug related rate × Group related rate × Referral related rate*

*Based on these rates, even with the maximum level of insurance cover, patients still have to pay 50% of the cost of the IFN drugs and 70% of the cost of the Peg-IFN drugs (See Drug related rate within Figure 2) 32 . Furthermore, if patients attend the HTD without a formal referral from their primary health care facility, they have to pay for the full cost of the treatment (as though uninsured) (See Referral related rate within Figure 2 and Figure 3) ^33^ . The payment mechanism within the NHI is the same for the non-drug costs, but the drug related rates are superseded by the relevant non-drug rates (see equation 3).”*

 The co-payment mechanism of the non-drug costs is the same but with different rates. We used the drug cost as an example to show the general idea of how national health insurance works. We have made this clearer in the text.

 Minor changes including editorial points:

 Introduction first paragraph contains an extra close brackets at the end of line 7.

**Response:** We have deleted the blanket.

 Methods first paragraph refers to gross per capita income but cites a source of GDP per capita. Clarify if referring to GDP, GNI, or per capita income which is referred to in discussion with a different source cited.

**Response**: We have fixed the references for this and changed the values to GDP per capita.

 Table 3 footnote refers to itself, should this refer to Table 4?

**Response:** We have fixed this (changing Table 3 to Table 4).

 Table 4, please indicate which class of drugs each brand name refers to so that it can be cross-referenced with Table 3.

**Response**: We have fixed by adding subcategory for column 1 within Table 4.

 Figure 3 caption refers to itself, should this refer to Figure 2?

**Response**: We have fixed this (changing Figure 3 to Figure 2).

 The reference for the cost of DAAs is fairly old, unfortunately there is not much published on this but MSF's press release on what price they pay may be helpful.

**Response:** We have updated the references related to the cost of DAAs

*“It has been estimated that widespread access to combinations of HCV DAAs is feasible, with potential target prices approximately US$50–$250 per person for a standard 12-week treatment course, significantly cheaper than the longer treatment regimens with IFN and Peg-IFN [REF 1-2]”*

**REF 1:** Hafez TA. Public Health and Economic Burden of Hepatitis C Infection in Developing Countries.  Hepatitis C in Developing Countries: Elsevier; 2018. p. 25-32.

**REF 2:** Barber MJ, Gotham D, Khwairakpam G, Hill A. Price of a hepatitis C cure: cost of production and current prices for direct-acting antivirals in 50 countries. Journal of Virus Eradication. 2020.

 Discussion states that cost data related to IFN supports economic case to increase access to DAAs. Perhaps rephrase to indicate that the cost data provides a foundation for evaluating the economic case to treat with DAAs, as the case for use of DAAs is not made in this paper. Or remove as this is stated later in discussion as well (bottom of page 9 column 1).

**Response:** Thank you for this suggestion. We have rephrased the statements in the discussion and conclusion.

*“This cost data related to interferon-based therapy indicates that switching to generic DAAs may lead to potential cost savings”*

*“Although the role of interferon-based therapy is diminishing, this cost data provides a foundation for evaluating the economic benefits and cost-effectiveness of switching to DAAs*.”
